# Elasticity-induced force reversal between active spinning particles in dense passive media

**DOI:** 10.1038/ncomms11325

**Published:** 2016-04-26

**Authors:** J. L. Aragones, J. P. Steimel, A. Alexander-Katz

**Affiliations:** 1 Department of Materials Science and Engineering, Massachusetts Institute of Technology, Cambridge, Massachusetts 02139, USA

## Abstract

The self-organization of active particles is governed by their dynamic effective interactions. Such interactions are controlled by the medium in which such active agents reside. Here we study the interactions between active agents in a dense non-active medium. Our system consists of actuated, spinning, active particles embedded in a dense monolayer of passive, or non-active, particles. We demonstrate that the presence of the passive monolayer alters markedly the properties of the system and results in a reversal of the forces between active spinning particles from repulsive to attractive. The origin of such reversal is due to the coupling between the active stresses and elasticity of the system. This discovery provides a mechanism for the interaction between active agents in complex and structured media, opening up opportunities to tune the interaction range and directionality via the mechanical properties of the medium.

## Abstract (web summary)

Physics out-of-equilibrium is necessary to understand a variety of interactions, for example in biological systems. Here, the authors show that the interactions between non-Brownian active spinning particles can change from repulsive to attractive depending on the properties of the surrounding passive medium.

## Graphical abstract

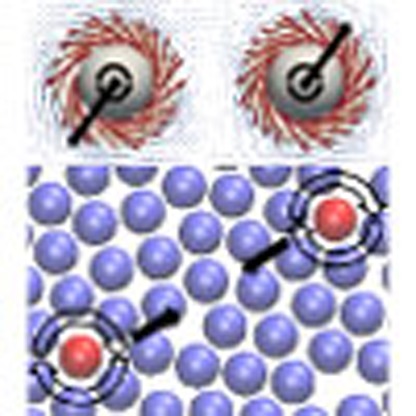

Life occurs out of equilibrium. Living organisms are continuously generating and consuming energy to achieve self-generated motion. In addition, equilibrium conditions are barely found in nature or industrial material processing. Thus, due to their out-of-equilibrium nature, active systems have attracted much attention in recent years. These systems exhibit exotic behaviours not possible under equilibrium constraints such as emergent collective motion^1^,^2^, pattern formation^3^,^4^,^5^ or even phase segregation in the absence of attractive interactions^6^,^7^,^8^,^9^. The most studied active agents are those which convert some sort of energy into translational motion. Such systems resemble how bacteria swim, and are known as self-propelled agents^6^,^7^,^10^,^11^. Energy conversion into rotational motion is also common in nature; important examples are the motor adenosine triphosphate synthase^12^, certain cilia^13^, the vortex array formation of sperm cells^3^ and the dancing Volvox^14^. Furthermore, experiments and several numerical and theoretical studies have focused on this type of active system in viscous media^15^,^16^,^17^,^18^,^19^,^20^,^21^,^22^,^23^,^24^,^25^. In addition to the type of activity, the medium can have a great influence on the effective interaction between different particles, which can be particularly important for active systems. Mixtures of active and passive particles can be used as a model system where active particles are embedded in a complex passive system, a scenario that is prevalent in many biological systems or processes. For example, bacterial biofilms, where live and dead bacteria phase segregate^26^,^27^, cell migration through tissues^28^,^29^ or sperm swimming through the viscoelastic cervical mucus^30^. Although these systems are ubiquitous, very few works have investigated these hybrid active–passive matter systems^2^,^31^,^32^,^33^,^34^,^35^,^36^. McCandlish *et al*.^31^ reported phase segregation of active rods in the presence of passive rods, where they point to a dynamical instability as the origin of activity-induced phase segregation; this instability originates from the differential parallel and transversal diffusion coefficients coming from the anisotropy of the rods. Ni *et al*. focused on the behaviour of a passive particle suspension in a glassy state doped with active agents. They observed that the presence of active particles shift the glass transition toward higher packing fractions^32^ and promote the crystallization of hard-sphere glasses^37^. Stenhammar *et al*.^34^ showed that mixtures of self-propelled and passive particles phase separate into a dense and a dilute phase, between which the interfacial tension is negative^38^. However, despite all these efforts, the origin of the emergent interactions between active agents in mixtures with passive agents remains unclear.

To shed light on the emergent interactions that govern the self-organization of non-Brownian active rotating particles, henceforth referred to as spinners, in systems composed of mixtures of active and passive particles, we use both experiments and simulations. We focus on the behaviour of pairs of co-rotating and counter-rotating spinners suspended in a viscous fluid or embedded in dense monolayers of passive particles. Importantly, we show a force reversal between spinners as the concentration of passive particles increases above a threshold. In particular, we observe that in a viscous fluid at small but finite Reynold numbers (Re), the fluid flows generated by co-rotating spinners produce a repulsion between spinners (Fig. 1a), whereas for counter-rotating spinners the resulting forces are attractive (Fig. 1b). By contrast, two co-rotating spinners in a dense passive monolayer attract each other (Fig. 1c), whereas counter-rotating spinners repel (Fig. 1d). We demonstrate that this force reversal is induced by the change in the mechanical properties of the matrix, from a viscous medium, if suspended in the fluid, to a solid-like viscoelastic medium, in the presence of passive particles. We anticipate that this mechanical attraction between co-rotating spinners is responsible for the phase separation between active and passive particles in macroscopic systems.

## Results

### Spinners in a viscous fluid

Our experimental system is composed of spherical ferromagnetic particles of diameter *σ* coupled to an external rotating magnetic field of frequency *ω*, see Supplementary Fig. 1. For the experimental conditions, the rotational frequency of the particles always coincides with the rotational frequency of the field. Spinners suspended in an incompressible fluid (∇***u***=0) of viscosity *η* and density *ρ* generate fluid flows that can be described by the Navier–Stokes equation:


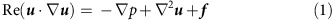


where we have assumed no-slip boundary conditions at the particle surface, ***u***=***U***+***ω*** × ***r*** being *U* the translational velocity of the particle, *ω* the angular velocity and ***r*** the vector pointing from the center of mass of the particle to the surface of the particle. In equation (1), we have chosen a translating reference frame at the center of the spinner (that is, such as the flow is steady) and scaled the velocities and lengths by *ωσ* and *σ*, respectively. ***u*** corresponds to the fluid velocity field, Re is the Reynold number (Re=*ωσ*^2^*ρ*/*η*), *p* is the pressure and ***f*** is the force density exerted by the particle on the fluid. Therefore, in the absence of any other particle, and in the limit of Re=0 (that is, where the left-hand terms in equation (1) are 0), a rotating spherical particle generates a velocity field given by ref. 39.


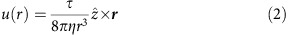


where ***r*** is the position of the fluid from the center of the particle, 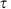 is the torque acting on the particle, and 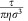 corresponds to the angular rotational frequency (*ω*) of the spinner, which is constant in our system. This rotating field decays as 1/*r*^2^ from the center of the spinner, as shown in Supplementary Figs 2–4 and Supplementary Discussion.

**Figure 2:**
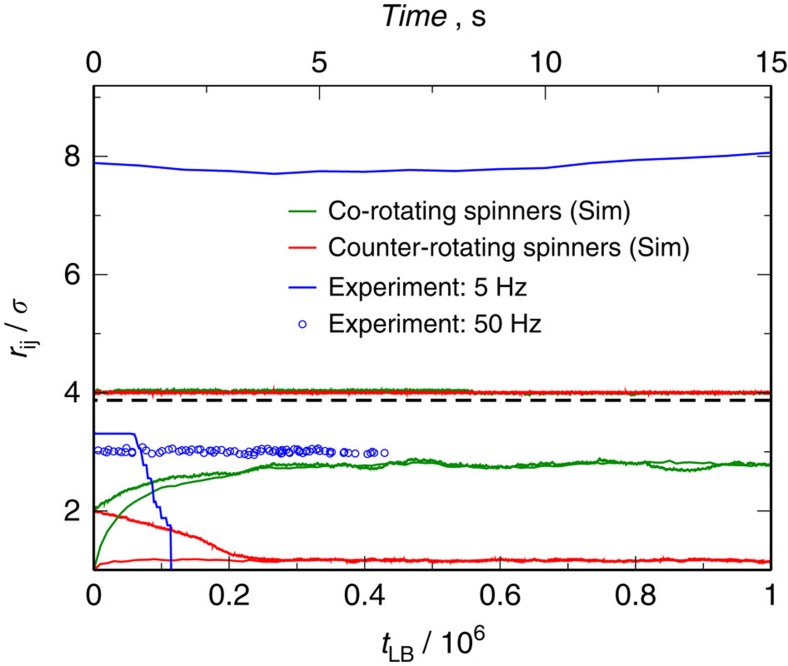
Evolution of the distance between spinners in an incompressible fluid. Time evolution of the distance between two co-rotating, *r*_*ij*_, in the experiments at 5 Hz (Re=1.25 × 10^−3^) at two different initial distances, blue lines. The blue circles corresponds to the trajectory at 50 Hz (Re=1.25 × 10^−2^). Time evolution of the distance between two co-rotating in our simulation model for two different initial distances, green lines, at Re=0.84; and between two counter-rotating spinners at two different initial distances, red lines, using simulations at Re=0.84. The dashed line at 4*σ* points out the experimental threshold for the attraction between spinners suspended in an incompressible fluid. The spinners are on the bottom wall of a channel of height *h*=30 Δ*x*.

First, we study the effective interaction between two spinners in a dilute system of co-rotating spinners in the absence of passive particles, see Supplemetary Methods. In this system, the interaction between spinners is controlled by the fluid flows generated by the rotation of the spinners and the magnetic dipole–dipole attraction between them. Experimentally, we observe that spinners initially positioned further than four particle diameters do not feel either the fluid flow created by other spinners or the permanent dipole of the other spinners. Therefore, they rotate in place without experiencing any translation, as shown in Fig. 2. By contrast, spinners closer than 4*σ* attract due to the magnetic dipole–dipole interaction, thereby forming a doublet and rotating around its center of mass. To get a deeper insight in the behaviour of the system, we also perform hybrid molecular dynamic simulations of the spinners sedimented onto a wall within a channel of height *h*=30 Δ*x* and coupled to a Lattice–Boltzmann (LB) fluid^40^. These simulations, which lack the dipole–dipole interaction, show that co-rotating spinners closer than 3*σ* experience a hydrodynamic repulsion, while rotating around the center of mass of the repulsive pair. We hypothesize that in our experiments, the hydrodynamic repulsion is hidden by the strong dipole–dipole interaction between ferromagnetic particles. To test this, we increase the rotation frequency of the applied magnetic field up to 50 Hz. At this frequency the hydrodynamic repulsion overcomes the dipole–dipole attraction, and spinners separate up to 3*σ*, blue circles in Fig. 2. The fact that the hydrodynamic repulsion increases with Re proves the inertial nature of the interaction^15^, and it is in good agreement with previous observations on millimeter-sized rotating magnetic disks adsorbed at the air–water interface^15^,^16^. Although our experimental set-up does not provide us with control over the direction of rotation of individual spinners, our simulations allow us to explore the case of spinners rotating on opposite directions. In this case, we find that two counter-rotating spinners closer than 3*σ* attract each other until the separation distance between them becomes about 1.15*σ* (see Supplementary Fig. 5), and simultaneously translate as a doublet in the direction orthogonal to the vector joining both centers^41^. The equilibrium distance between counter-rotating spinners does not depend on the Re, but the strength of the interaction and translational velocity of the center of mass does (see Supplementary Fig. 5 and Supplementary Discussion), which indicates that the observed attraction between counter-rotating spinners is also inertial in nature.

**Figure 3:**
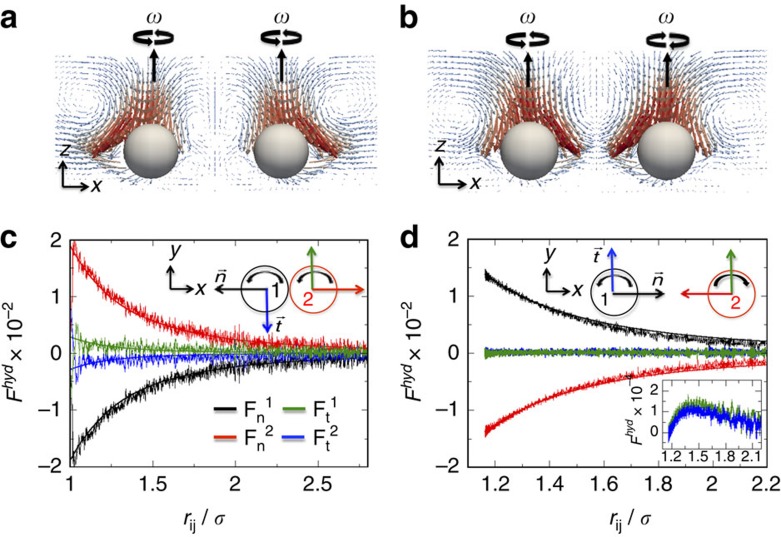
Interactions between spinners in a viscous fluid. Polar flow fields generated by two co-rotating spinners (**a**) and by two counter-rotating spinners (**b**) that have sedimented onto a wall within a channel of *h*=30·Δ*x*. Hydrodynamic forces acting on each spinner as a function of the distance between them for two co- (**c**) and counter-rotating spinners (**d**). Solid lines are fits to the data with the function *a*_0_/*x*^3^.

Inertial contributions to the fluid velocity field, left-hand terms in equation (1), are the origin of the repulsion between co-rotating spinners^17^,^42^ and the attraction between counter-rotating spinners. At a finite Re, inertial terms generate additional forces on the particles due to the momentum of the fluid. These type of forces, known as lift forces, originate from the relative translation of a rotating particle with respect to the fluid^43^, known as Magnus forces, or by the translation of the rotating particle with a shear flow^44^. Both lift forces depend on the translational velocity of the rotating particle. Under these conditions, the fluid velocity profile generated by a rotating sphere, equation (2), needs to be corrected to include these inertial terms, which generate a so-called secondary flow. Perturbation methods have been used to calculate the secondary flow around a rotating sphere due to small inertial effects^45^; these studies have shown that the secondary flow produces no correction in the azimuthal part of the fluid velocity profile, equation (2). However, because of the centrifugal force effect, the fluid is pulled in towards the poles and expelled from the equator, which generates a secondary flow on the *zx*-plane (see Supplementary Fig. 6). The presence of a second rotating sphere breaks the symmetry of the secondary flow; around the equator of the spheres the fluid velocity between the spinners decreases for co-rotating spinners and increases for counter-rotating spinners, as shown in Fig. 3a,b. We compute the forces exerted by the fluid on co- and counter-rotating spinner pairs along trajectories of repulsion and attraction, as shown in Fig. 3c,d, respectively. For two co-rotating spinners, hydrodynamic forces generate a net repulsion between them, while a hydrodynamic attraction is generated for the case of counter-rotating spinners. The fluid flows generated by two co-rotating spinners cause the spinners to rotate around their center of mass. This translation of the spinners generate a lift force that results in a repulsion of co-rotating spinners^17^,^18^. Previous studies have shown that both co-rotating and counter-rotating spinners, repel each other as a consequence of the lift forces^42^; however, in the presence of a channel the hydrodynamic attraction between two counter-rotating spinners overcome the lift force, resulting in an effective attraction^25^. As discussed below, the equilibrium distance between counter-rotating spinners does not depends on the Re number (Supplementary Fig. 5); however, it does on the channel height (Supplementary Fig. 7 and Supplementary Discussion). Thus, the confinement of the counter-rotating pair reduces the strength of the lift forces, making the hydrodynamic attraction dominant. Similarly, the confinement of co-rotating spinners pairs results in a shorter repulsion distance (Supplementary Fig. 7) because of the reduction of the lift force magnitude^25^.

### Spinners in a dense passive media

**Figure 4:**
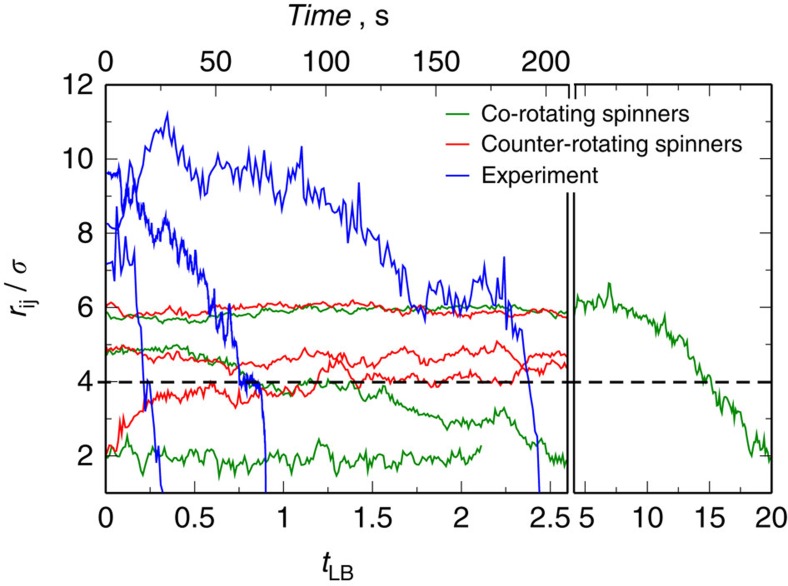
Evolution of the distance between spinners embedded in a passive particle monolayer. Time evolution of the distance between two co-rotating spinners in the experiments at Re=1.25 × 10^−3^ (blue lines) and in our simulation model at Re=0.84 (green lines); and between two counter-rotating spinners (red lines) at Re=0.84 using simulations. In the simulations the monolayer area fraction is *ϕ*_A_=0.8, whereas in the experiments is of about *ϕ*_A_=0.7±0.1. The dashed line at 4*σ* points out the experimental interaction threshold between spinners suspended in an incompressible fluid. The spinners are on the bottom wall of a channel of hight *h*=30·Δ*x*.

The behaviour of spinners embedded in monolayers of passive particles is completely different. At finite Re, the fluid flow generated by a spinner repels neighbouring passive particles, and generates the rotation of the first shell of particles around it (see Supplementary Movies 1 and 2). The distance of this first shell of passive particles with respect to the spinner depends on the area fraction of the monolayer, *ϕ*_A_, and the angular rotational frequency of the spinner, *ω*. Therefore, the spinner produces a local increase of the mobility of neighbouring passive particles and compresses the monolayer (Supplementary Fig. 8). When more than one spinner are present in the monolayer, we observe that two co-rotating spinners attract each other; this behaviour is opposite to that observed in the absence of passive particles, as shown in both experiments and simulations in Fig. 4. The experimental trajectories of two co-rotating spinners in a monolayer with an area fraction of *ϕ*_A_=0.7±0.1 show two well-differentiated regimes: (i) If the distance between the spinners is smaller than 4*σ*, then the slope of the trajectory is sharp; this indicates that the attraction between spinners in this regime is governed by the strong magnetic dipole–dipole interaction. (ii) At distances larger than 4*σ*, the slope is small, which indicates that the attraction between spinners must be of a different nature. By contrast, in the simulations the trajectories exhibit a single regime of slow attraction due to the lack of dipole–dipole interactions in our model. Furthermore, once the spinners squeeze out all the passive particles initially positioned between them, they remain as a doublet at a distance of about 2*σ* for monolayers of *ϕ*_A_=0.8. In our experiments, we can only study co-rotating spinners, a limitation absent in our simulations. Thus, using simulations we find that two counter-rotating spinners repel each other at a distance of about 5*σ* within a monolayer of *ϕ*_A_=0.8. Therefore, we also observe a reversal of the interaction force between two counter-rotating spinners with respect to the pure viscous media in the presence of a passive matrix.

**Figure 5:**
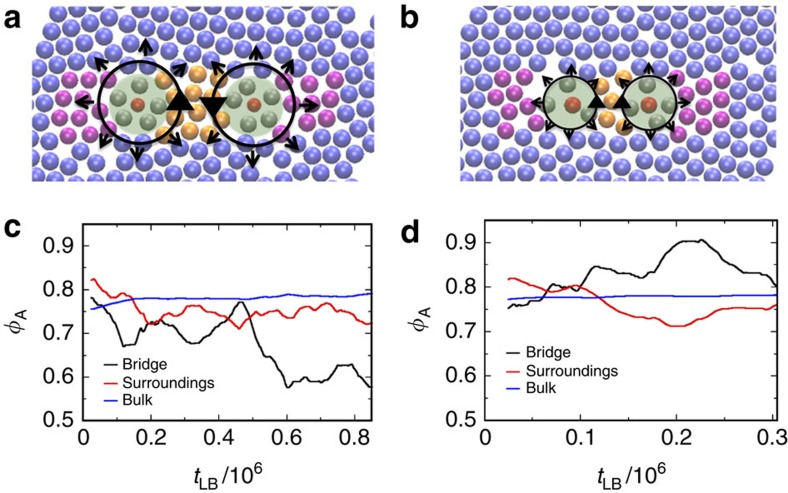
Effect of the spinners in a passive monolayer of *ϕ*_A_=0.8. (**a**,**b**) Illustration showing the forces on the system and the different regions we define: in green shade the corona, which includes the spinner (red sphere) and the particles around it (grey spheres), the bridge, particles located between the spinners (yellow spheres), the surroundings, particles located besides the spinners on the opposite side of the bridge (purple spheres) and the bulk (blue spheres). (**c**) Time evolution of the particle area fraction of the bridge (black line), the surroundings (red line) and the bulk (blue line) for co-rotating spinners separated by 6*σ*. (**d**) Time evolution of the particle area fraction of the bridge (black line), the surroundings (red line) and the bulk (blue line) for counter-rotating spinners separated by 4*σ*. The position of the spinners is frozen.

To investigate the nature of the interaction between two spinners in the presence of the passive matrix, we define four different regions in the system and label the particles within these regions accordingly. These four regions are: the first shell of particles around the spinners, named as corona, the region between the two spinners, referred as bridge, the region besides the spinners on the opposite side of the bridge, denoted as the surroundings, and the bulk, as illustrated in Fig. 5a,b. Particles located in the corona rotate coherently around the spinners, and collide against neighbouring particles transferring their momentum. These particles rarely escape from this region and the number of particles in the corona remains almost constant until the coronas of the two spinners starts to collide with each other. Therefore, we count these particles as a part of the spinner for every calculation (green shade region in Fig. 5a,b). The bridge and the surroundings are very dynamic; particles in these regions are in continuous motion. The stresses generated by the spinners through the corona are released in these regions. To relax the stresses they need to yield.

To study the evolution of the passive particles in the bridge, surroundings and bulk decoupled from the spinners rearrangement, we perform numerical simulations freezing the distance between the spinners at different values. These constrained systems are just able to relax the stress coming from the activity of the spinners through the displacement of passive particles, and thus they never reach a steady state. However, the initial time evolution of these systems allows us to study the process of loading and yielding of the monolayer without the relaxation of the system through the displacement of the spinners. In Fig. 5c,d, the initial time evolution of the particle area fraction in the different defined regions are presented for co-rotating and counter-rotating spinners separated 6*σ* and 4*σ*, respectively. We observe that for co-rotating spinners, the *ϕ*_A_ of particles in the bridge is significantly reduced as compared with the *ϕ*_A_ in the surroundings and bulk, as shown in Fig. 5c and Supplementary Fig. 9. The compression and shear stresses produced by the spinners in the bridge, through the corona, result in a *ϕ*_A_ reduction within this region as it is constantly yielding due to shear stresses induced by the spinners. Thus, the higher mobility of passive particles initially located in this region allows them to migrate to less-stressed regions. On the contrary, for counter-rotating spinners, the *ϕ*_A_ of passive particles in the bridge is significantly increased compared with the bulk and surroundings, as shown in Fig. 5d and Supplementary Fig. 9. Therefore, the mechanism by which the passive matrix mediates the interaction between spinners is related to the type of stresses that the spinners exert on their vicinity. Co-rotating spinners compress and shear the bridge, as schematically illustrated in Fig. 5a. To alleviate the stress, the system prefers to yield by transporting particles from the bridge into the other regions. This occurs through avalanches and single-particle hopping, as will be shown later. Clearly, this migration reduces the density on the bridge. This imbalance repositions the spinners closer to each other, thereby restoring the temporal mechanical equilibrium. This process is continuously occurring, which slowly degrades the bridge until the active particles are able to come together (Supplementary Movies 1 and 2). During this process the monolayer is annealed, inducing the defects to migrate and concentrate around the spinners, as depicted in Supplementary Fig. 10 and Supplementary Discussion. We discard the migration and coalescence of defects as the driving force for the co-rotating spinners attraction by performing simulations in which the initial configuration is a perfect hexagonal close packing lattice. In this case, co-rotating spinners aggregation is still observed, as shown in Supplementary Fig. 11. By contrast, counter-rotating spinners produce compression and dilation stresses in the bridge. Both spinners move passive particles into the bridge, which increases the pressure in this region; this pushes both spinners away, thereby resulting in a repulsion between counter-rotating spinners (Supplementary Movie 3).

**Figure 6:**
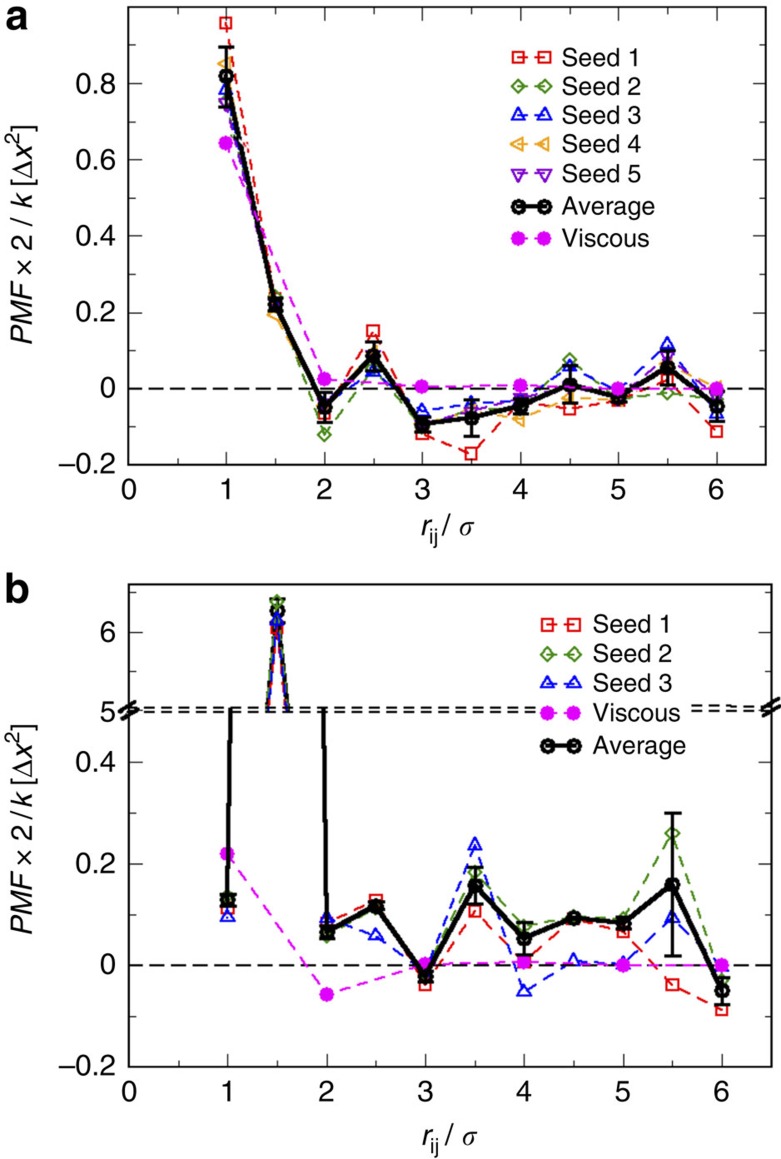
Spinner–spinner interaction within passive monolayers of *ϕ*_A_=0.8. (**a**) Work done by the springs as a function of the separation distance between two co-rotating spinners. The black circles represent the average potential of mean force, PMF, normalized by the spring constant, *k*, over five independent initial configurations of the spinners within the monolayer: Seed 1 (red squares), Seed 2 (green diamonds), Seed 3 (blue up triangles), Seed 4 (orange left triangles) and Seed 5 (violet down triangles). The error bars correspond to the s.d. of the mean over the five independent initial configurations. The magenta solid circles represent the PMF in the case of spinners suspended in a viscous fluid, that is in the absence of passive particles. (**b**) PMF as a function of the separation distance between two counter-rotating spinners. The black circles represent the average work over three independent initial configurations of the spinner within the monolayer: Seed 1 (red squares), Seed 2 (green diamonds) and Seed 3 (blue up triangles). The magenta solid circles represent the PMF in the case of spinners in a viscous fluid. The error bars correspond to the s.d. of the mean over the three independent initial configurations.

To further investigate this emergent interaction between spinners embedded in passive matrixes, we estimate the mean spinner–spinner interaction potential in monolayers of *ϕ*_A_=0.8, as shown in Fig. 6. By means of the application of harmonic springs, *F*=*k*(*r*−*r*_0_), to restrain the separation distance between the spinner pair at *r*_0_, we calculate a potential of mean force between co- and counter-rotating spinners, as presented in Fig. 6a,b, respectively. The mean interaction potential, black circles, is obtained by averaging over five independent initial configurations at each separation distance *r*_0_. The emergent interaction between two co-rotating spinners within a passive monolayer is repulsive at distances smaller than 2*σ*. At larger distances, shallow attractive minima are separated by small energy barriers, as shown in Fig. 6a. The local energy input coming from the spinners' activity is enough to overcome these small energy barriers. The presence of these energy barriers at distances larger than 2*σ* agree with the observation that the loading of the bridge plays a key role for this interaction. Moreover, the shallow minima would then correspond with the state of the system after an yielding event. In the case of counter-rotating spinners, the interaction is repulsive with a strong repulsive peak at 1.5*σ*; it also presents a small energy minimum at 3*σ*, as can be seen in Fig. 6b. This shallow energy minimum explains that along the repulsive trajectories, the spinners spend long times at this distance, as it can be seen in Supplementary Fig. 12B. We also use this methodology to evaluate the interaction potential between co- and counter-rotating spinners in the absence of passive particles, represented by the magenta solid circles in Fig. 6a,b, respectively. In agreement with our previous calculations, Fig. 6 clearly shows that the change in the mechanical properties of the media produces not only a reversal of the forces acting on the spinners but also significantly increases the range of the interaction. Interestingly, the potential of mean force at the different separation distances strongly depends on the initial configuration of the monolayer, as shown in Fig. 6; the average s.d. between the different initial configurations is about 10%. Therefore, in spite of the general trend captured by these mean spinner–spinner interaction potentials, this interaction is of a stochastic nature and thus, the strength and range of the interaction between spinners in passive matrixes is determined by the instantaneous and previous configurations of the monolayers, that is memory effects. Another proof of that is shown in Supplementary Fig. 13, where we compute, by means of harmonics springs that restrain the separation distance between co-rotating spinners, the attractive force between the spinners for 100 configurations selected from the unconstrained trajectory presented in Fig. 4 of spinners initially positioned 6*σ* apart, as shown in Supplementary Fig. 13A. The different configurations present significantly different values of the force, as it can be seen in Supplementary Fig. 13B; however, the average force as a function of the separation distance between the spinners is in good agreement with the one estimated by averaging over five independent configurations at each separation distance. We would like to point out that the direction of the interaction is not determined for a given distance (Supplementary Discussion; Supplementary Fig. 14). For example, in Fig. 6a at *r*_*ij*_=4.5*σ* the interaction can be either attractive or repulsive depending on the configuration (different seeds). Thus, for any spinner pair it is not possible to predict the exact local evolution, but only that they will attract or repel over the length of the experiment.

**Figure 7:**
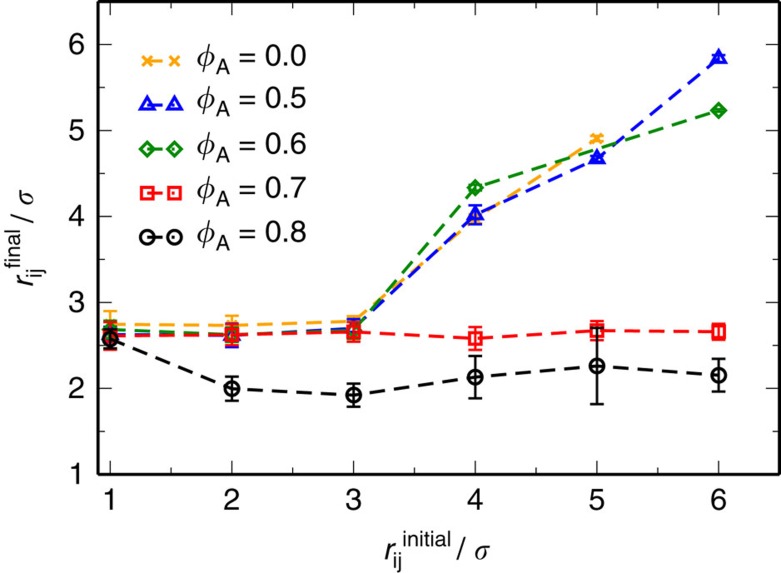
Co-rotating spinners in passive monolayers at different *ϕ*_A_. Initial distance between co-rotating spinners versus the final distance after a long simulation run at Re=0.84 in monolayers at different particle area fractions: *ϕ*=0 (orange crosses), *ϕ*=0.5 (blue triangles), *ϕ*=0.6 (green diamonds), *ϕ*=0.7 (red squares) and *ϕ*=0.8 (black circles). The error bars correspond to the s.d. of the average final distance between the spinners once they have reached each other.

**Figure 8:**
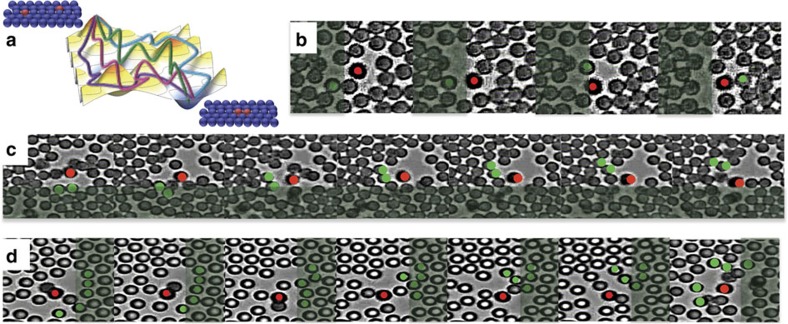
Bridge erosion mechanisms. (**a**) Sketch illustrating the rough energy landscape the spinners have to move through to form a dimer. Three different mechanism by which the passive particles are squeezed out from the bridge by the stress imparted by the spinners: (**b**) A single passive particle from the bridge jumps into the corona of one of the spinners and is released in the surroundings or the bulk. (**c**) Multiple particle removal mechanism: Two or three passive particles are taken from the bridge and moved to the bulk or surroundings. (**d**) Avalanche mechanism: an entire shell of particles is removed from the bridge. The shaded regions indicate the bridge.

We have also found in both experiments and simulations that the aggregation process of co-rotating spinners embedded in a monolayer is governed by the elasticity of the medium and the ability of the spinners to increase the elastic energy of the system. This can be directly confirmed by measuring the storage and loss moduli of the system in the absence of active particles, and observing at what particle area fraction the attractive interaction between co-rotating spinners is lost (Supplementary Discussion; Supplementary Figs 15 and 16). In Fig. 7, the initial distance between two co-rotating spinners is represented against the distance reached after a long run. For passive monolayers at *ϕ*_A_=0.8 and 0.7, spinners initially separated up to 6*σ* are attracted to each other up to a distance of about 2*σ* and 2.5*σ*, respectively. However, for particle area fractions smaller than 0.7, passive-mediated interactions are no longer effective and only the hydrodynamic repulsion is observed. According to the mechanical properties of the passive monolayer, the elastic response of the system dominates at long times for monolayers of *ϕ*_A_=0.8 and 0.7, as depicted in Supplementary Fig. 16A,B; for the latter, elastic and viscous responses are almost equivalent at long times. On the contrary, for area fractions of 0.6 and 0.5 the viscous response dominates for the entire frequency range, Supplementary Fig. 16C,D. This demonstrates that an elastic response of the passive media is a necessary condition for co-rotating spinners to interact. As mentioned before, this elasticity-mediated attraction between active spinning particles is inherently stochastic. Thus, the initial configuration of the system determines the length of interaction and the time required for spinners to aggregate, as shown by the different experimental and simulation trajectories in Fig. 4. Hence, one can imagine a rough and dynamic energy landscape in which the spinners must traverse to form a dimer, and where multiple paths exist when moving from state to state, as schematically illustrated in Fig. 8a. Erosion of the bridge, which happens when passive particles are removed from it, occurs through three main mechanisms: (i) Single-particle removal from the bridge to the corona and then to the bulk, as shown in Fig. 8b; (ii) multiple particle removal, where two or three particles in the bridge, due to the shear stresses, are moved into the bulk or intermittently moved to the corona and then ejected into the bulk, as seen in Fig. 8c; and (iii) avalanches where entire lines of particles in the bridge are pushed into the bulk by the shear exerted by the spinners, as depicted in Fig. 8d. We use the name avalanche to invoke the instantaneous and dramatic nature of the particle removal process. Whereas the bridge erosion produced by the two first mechanisms is slow and depends on stochastic collisions, the removal of particles in avalanche happens almost instantaneously. In fact, it has been previously shown that the devitrification of hard-sphere glasses is mediated by large rearrangements of particles, so-called avalanches^46^. This interaction opens up possibilities to study the mechanism of elasticity-mediated interaction between active particles in these systems governed by a glassy dynamics.

## Discussion

In summary, the forces exerted by an incompressible fluid at small but finite Re on a pair of non-Brownian active rotating particles depends on the relative sense of rotation of each particle, resulting repulsive for co-rotating spinners and attractive for counter-rotating spinners when confined in a channel. The presence of a dense passive matrix modify the mechanical properties of the system from a viscous media to a viscoelastic material. In this latter case, the interaction between spinners becomes controlled by elastic effects, which act in the opposite direction than inertial effects^47^,^48^,^49^. Hence, the switch between inertial and elastic stresses derives in a reversal of the interaction between spinners, resulting in an attraction of co-rotating spinners and a repulsion of counter-rotating spinners. In fact, the structure of the passive dense medium cannot be treated at the mean field level. For example, assuming a pure viscous scenario where the passive matrix would be an homogeneous continuum with higher viscosity than the system in the absence of passive particles, the stress would be dissipated by the viscous media, and one would just observe the repulsion between co-rotating spinners, but the strength of the secondary flows would be smaller due to the viscosity increment. Therefore, the change of the mechanical properties of the matrix from a viscous material to a solid-like material is the responsible for the force reversal. Furthermore, the interaction between spinners in dense monolayers of passive particles is of stochastic nature; it depends on the configuration of the passive monolayer. Thus, it is the instantaneous configuration of the monolayer that determines the strength and range of the interaction, and the dynamics of attraction is intimately related to the timescale for the rearrangement of the monolayer. This can be better understood by looking at the oscillation between periods of well-defined distances and periods of fast attraction along the trajectory between two co-rotating spinners, Figure 4 and Supplementary Fig. 12A. The level of stress put into the bridge by the spinners must reach a configuration dependent threshold value, and when this level is reached the system yields by removing entire groups of particles from the bridge, as depicted in Fig. 8. Then, the spinners approach each other and start stress loading the bridge again. Therefore, this effective interaction mediated by the passive medium cannot be seen as a position-dependent interaction potential (*U*(*r*_*ij*_)). Interestingly, the dynamic trajectories of the distance between the spinners initially positioned at different distances show an almost linear regime of attraction, as it can be seen in Fig. 4 and Supplementary Fig. 11. This means that the spinners approach each other on average at a constant speed. Assuming a Stokes' scenario, *F*=6*πμσ*/2*U*, for the translation of the spinners through the monolayer along their attractive trajectory, the strength would be a constant and independent on the distance between them. Moreover, this elasticity-mediated interaction is of a very long range. For example, in our simulations we observe that spinners separated up to six-particle diameters still interact, whereas the hydrodynamic interaction reach only three-particle diameters. Remarkably, our experimental measurements show that the interaction threshold shifts even at longer distances, and spinners separated by up to 17*σ* attract each other^50^, while the dipole–dipole interaction reach 4*σ* (Figs 2 and 4). Our results resemble other elastic media such as lipid membranes, where elasticity-mediated forces between transmembrane embedded proteins show logarithmic decays^51^,^52^. However, the origin of the stresses in our system are different.

In conclusion, we have shown that the interaction between active spinning particles depends on the properties of the medium and the dynamics of the active particles. Therefore, this cooperative interaction between the mechanical and dynamical properties of the system offers a variety of possibilities to tune this type of interaction between active agents. Remarkably, we have also observed that the spinners produce an annealing of the passive matrix structure (Supplementary Fig. 10), in agreement with previous simulation results in hybrid passive–active systems but with a different type of active particles^33^. We anticipate that this mechanical attractive force between co-rotating spinners is responsible for the phase separation observed in systems with higher concentrations of spinners. Moreover, in ternary hybrid active–passive systems, composed of mixtures of spinners rotating either clockwise or counter clockwise, we observe phase segregation in three different phases: one composed of the passive particles, other of co-rotating spinners, and the last one of counter-rotating spinners. In principle, this elasticity-induced interaction between active spinning particles is general for other active agents such as self-propelled particles. Therefore, this study opens up routes to control the range and direction of the interaction between active units in passive and structured environments. This interaction between active spinning particles in passive matrixes is different from the emergent interactions observed between passive objects within active fluids. Those effective interactions mediated by active matter between passive objects depend on the mobility of the passive objects^53^,^54^ and their shape^55^. Therefore, it would be very interesting to investigate these effects in the opposite scenario between active particles in passive matrixes (Supplementary Fig. 17). Even more, this type of interaction could play an important role in overcoming diffusive limitations that active biological molecules encounter if interacting within dense viscoelastic materials such as the highly viscoelastic nucleus of the cell^56^ or cells in extracellular polysaccharide matrix secreted by biofilm-forming bacteria^57^.

## Methods

### Experimental set-up

To study the behaviour of active rotating particles in pure viscous and in dense passive environments, we have designed a hybrid active–passive system composed by ferromagnetic particles (the active units or spinners) and polystyrene particles (the passive units). To measure pairwise interactions between spinners we mix an extremely dilute solution of active ferromagnetic particles with a solution containing passive polystyrene particles. Both particles have a diameter of 5 μm. We first study the case of pure spinners, in the absence of any passive particle. We then study the interactions between spinners in a monolayer of passive particles at a particle area fraction of *ϕ*_A_=0.7±0.1. These solutions, dilute spinners and dense mixtures, are placed between a cover slip and a slide, and placed in our experimental set-up, which is composed by four magnetic coils (Supplementary Fig. 1); this allows us to generate a rotating magnetic field that is parallel to the substrate and rotates around the *z* axis (see Supplementary Information for more details). The frequency of rotation used in this study was 5 Hz. On actuation of the external rotating magnetic field the magnetic dipole moment of the spinner couples to the applied field, and the particle spins in place around the *z* axis.

### Simulation

To gain a more detailed insight into the non-equilibrium nature of this system we carry out numerical simulations using hybrid molecular dynamic simulations of the colloidal particles coupled to a LB fluid^40^. The simulation box is discretized in three-dimensional grids with resolution *N*_*x*_ × *N*_*y*_ × *N*_*z*_=214 × 214 × 30 bounded in the *z* direction by no-slip walls and periodic boundary conditions in the *x* and *y* directions. The LB fluid is described by the fluctuating LB equation^58^, which properly describes the dissipative and fluctuating hydrodynamic interactions. We implement the discrete 19-velocity model (D3Q19). The LB fluid parameters are density *ρ*=1, kinematic viscosity *ν*=1/6 and temperature *k*_*B*_*T*=2 × 10^−5^. For simplicity, we set the grid spacing Δ*x* and the LB time step Δ*t* equal to unity. Interactions between the LB fluid and the particles are described by the bounce-back rule^59^, and enforcing no-slip boundary conditions at the surface of the particles. Specifically, we implement the Aidun, Lu, Ding (ALD) method^60^, where particles are treated as real solid objects. Therefore, we do not taken into account lubrication forces; however, we tested that including lubrication forces only shifts towards smaller Re numbers (that is, smaller rotational frequencies) the behaviour observed here. In our simulation model, colloidal particles are considered as hard spheres^61^ of diameter *σ*=12Δ*x*; thus, we are just considering excluded volume interactions. The spinner activity is generated by imposing an external torque, 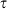, about the *z* axis. To form the monolayer we also include a gravity force *F*_G_=0.005. Since we are interested in the hydrodynamic interactions that occur between spinners, and not their magnetic interaction, we do not include dipole–dipole interactions in our simulation model to more clearly delineate the origin of the effective interactions. The typical simulation length is of about 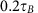, where 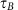 is the characteristic Brownian time.

## Additional information

**How to cite this article:** Aragones, J. L. *et al*. Elasticity-induced force reversal between active spinning particles in dense passive media. *Nat. Commun.* 7:11325 doi: 10.1038/ncomms11325 (2016).

## Supplementary Material

Supplementary Information Supplementary Figures 1-17, Supplementary Discussion, Supplementary Methods and Supplementary References

Supplementary Movie 1 Experimental trajectory of the attraction between two co-rotating spinners embedded in a passive monolayer of ΦA = 0.7.

Supplementary Movie 2 Simulation trajectory of the attraction between two co-rotating spinners embedded in a passive monolayer of ΦA = 0.8. The color labels are used to identify the particles located in the different regions of the system: Black particles are spinners, red particles locate within the bridge, white particles locate in the surroundings, pink particles locate in the corona and blue particles locate in the bulk

Supplementary Movie 3 Simulation trajectory of the repulsion between two counter-rotating spinners embedded in a passive monolayer of ΦA = 0.8. The color labels are used to identify the particles located in the different regions of the system: Black particles are spinners, red particles locate within the bridge, white particles locate in the surroundings, pink particles locate in the corona and blue particles locate in the bulk.

## Figures and Tables

**Figure 1:**
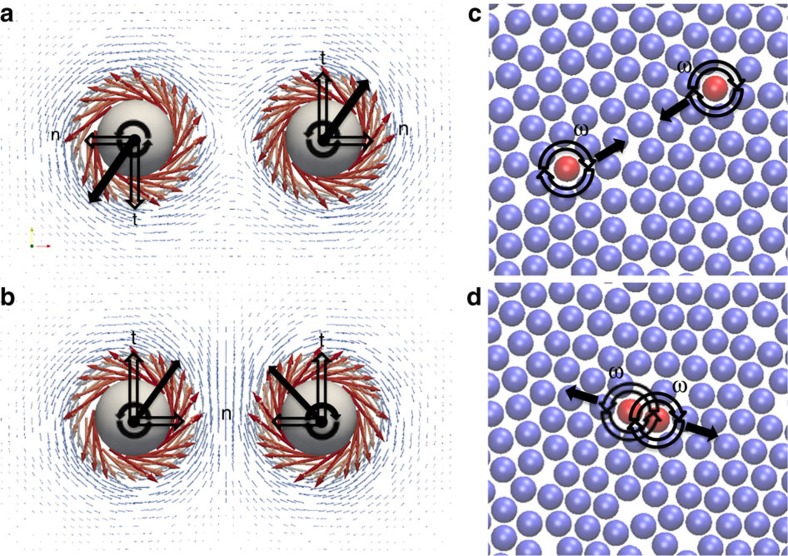
Schematic of the co- and counter-rotating spinners settled on a wall in a viscous fluid and embedded in a dense passive monolayer. (**a**) Fluid flows generated by two co-rotating spinners in a viscous fluid medium at finite Re result in spinner–spinner repulsion, as shown by the black arrows that indicate the direction of forces exerted by the medium on the spinners. The tangential components (t) come from the fluid flows generated by the neighbouring spinner, whereas the normal components (n) come from secondary flows. The resultant force generates trajectories where both spinners rotate around their center of mass while moving apart. (**b**) Fluid flows generated by two counter-rotating spinners at finite Re result in attraction. (**c**) Two co-rotating (red spheres) spinners rotating at frequencies *ω* in a dense monolayer of passive particles (blue spheres) attract. The effective forces exerted on the spinners by the passive medium are represented by black arrows. (**d**) Two counter-rotating spinners in a dense monolayer of passive particles repel (forces in black).
