# Supplementary material for: Elasticity-induced force reversal between active spinning particles in dense passive media
Source: Nat Commun. 2016 Apr 26;7:11325. doi: 10.1038/ncomms11325 (PMC4853433; doi:10.1038/ncomms11325)
Supplement: Supplementary Information — Supplementary Figures 1-17, Supplementary Discussion, Supplementary Methods and Supplementary References [file ncomms11325-s1.pdf]

I. SUPPLEMENTARY FIGURES

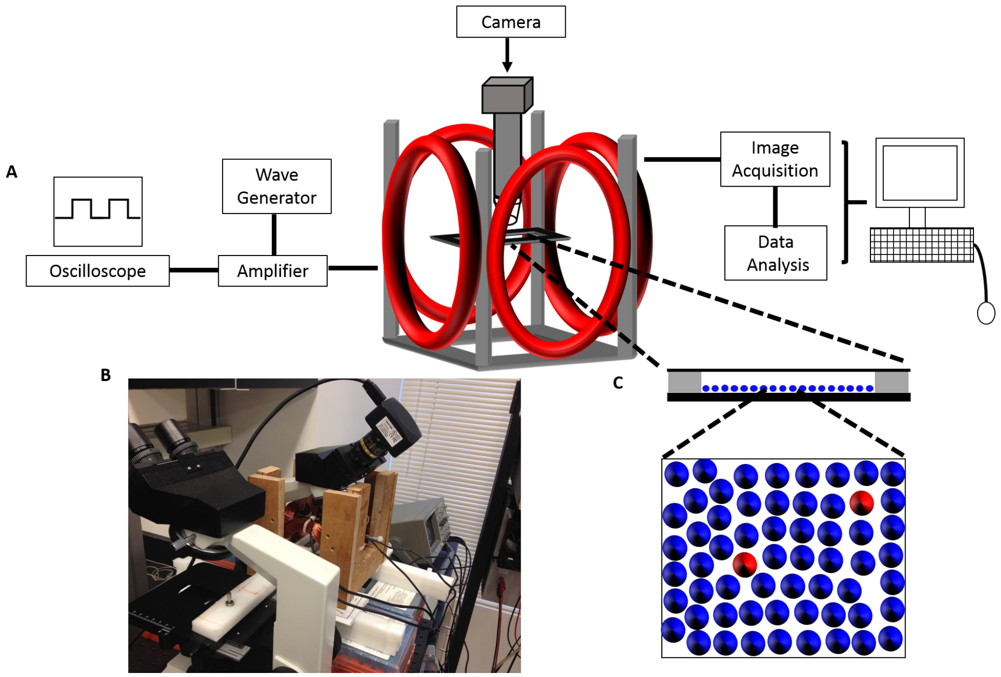

Supplementary Figure 1. Experimental Apparatus. A)Schematic of experimental set-up. A light microscope has been mounted with four coils to produce a rotating magnetic field. A wave generator ran two sinusoidal signals phase shifted by 90 degrees through an amplifier to generate a field strength of 5mT. An oscilloscope was used to monitor the frequency and a CCD camera mounted on the microscope was used to capture videos of the walker motion. B)Image of the experimental apparatus. C) The sample is placed at the center of the coils where the field is homogeneous. The solution of passive and active colloids in a surfactant solution is inserted into a channel and sealed.

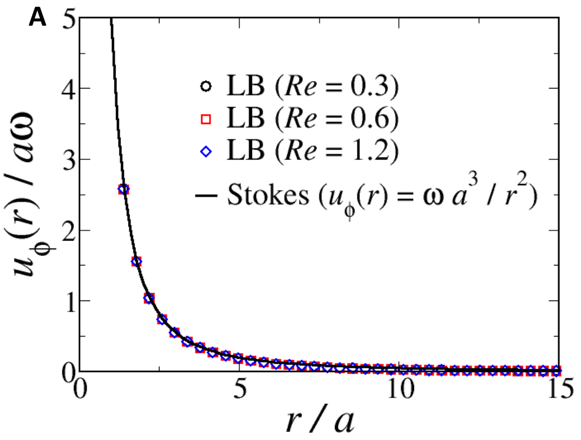

Supplementary Figure 2. Fluid flows in the absence of walls. A) Profile of the azimuthal velocity in the equatorial plane of a spinner at $Re = 0.3$ (black circles), 0.6 (red squares), and 1.2 (blue diamonds) in the absence of walls. The black solid line corresponds to the solution of the Eq. (2) in the main text. B) Profile of the polar velocity in the equatorial plane of a spinners at $Re = 0.3$ (black circles), 0.6 (red squares), and 1.2 (blue diamonds) in the absence of walls.

.

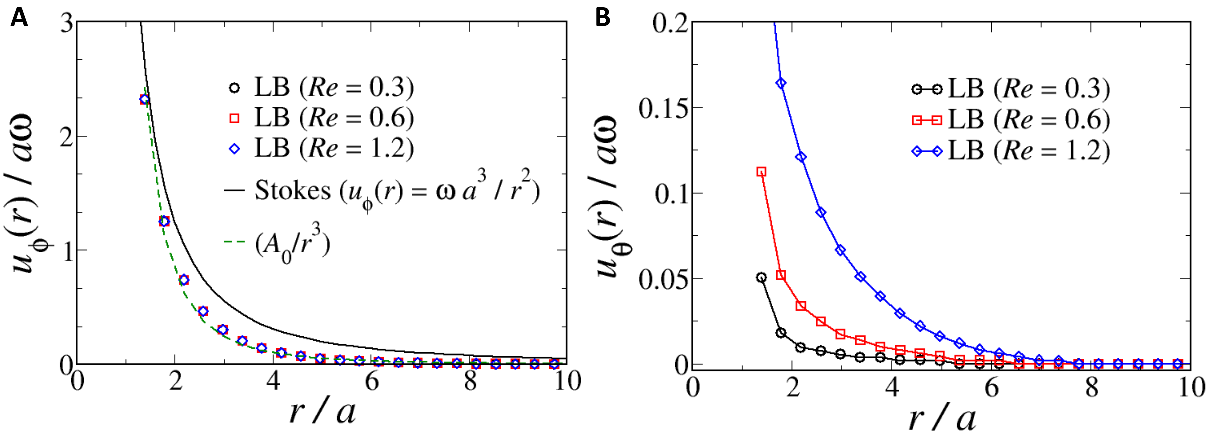

Supplementary Figure 3. Fluid flows in the presence of walls. A) Profile of the azimuthal velocity in the equatorial plane of a spinner at $Re = 0.3$ (black circles), 0.6 (red squares), and 1.2 (blue diamonds) at the bottom of a channel of height 30 Δx. The black solid line corresponds to the solution of the Eq. (2) in the main text. B) Profile of the polar velocity in the equatorial plane of a spinners at $Re = 0.3$ (black circles), 0.6 (red squares,) and 1.2 (blue diamonds) at the bottom of a channel of height 30 Δx.

.

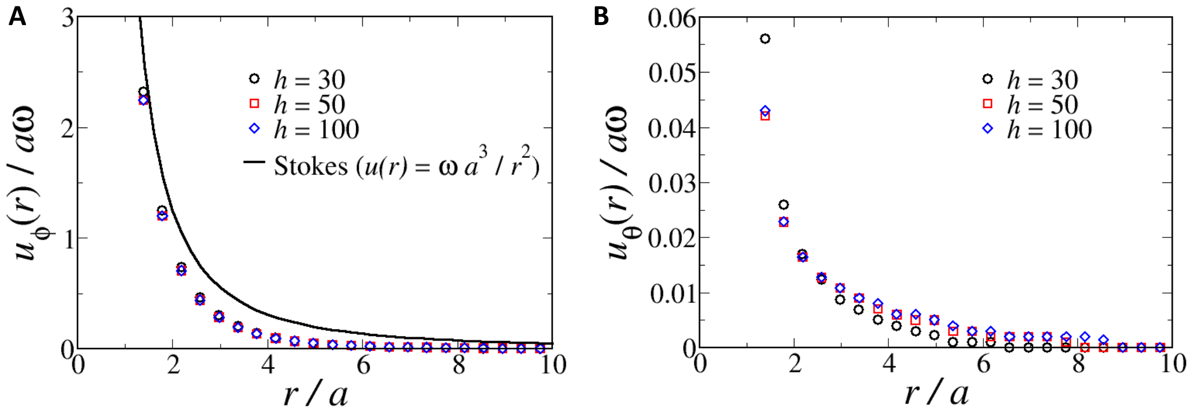

Supplementary Figure 4. Wall effects on the fluid flows. A) Profile of the azimuthal velocity in the equatorial plane of a spinner at $Re = 0.6$ in a channel of height $30\Delta x$ (black circles), $50\Delta x$ (red squares), and $100\Delta x$ (blue diamonds). The black solid line corresponds to the solution of the Eq. (2) in the main text. B) Profile of the polar velocity in the equatorial plane of a spinners at $Re = 0.6$ in a channel of hight $30\Delta x$ (black circles), $50\Delta x$ (red squares), and $100\Delta x$ (blue diamonds).

.

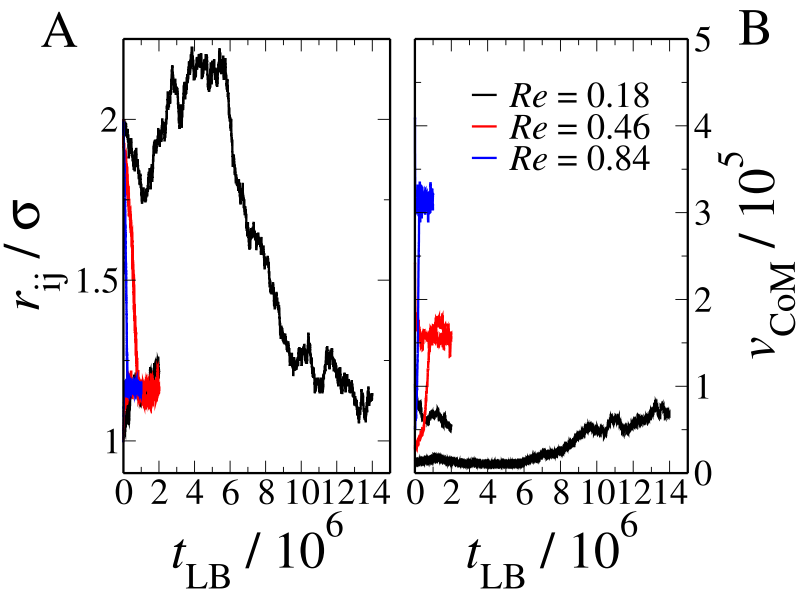

Supplementary Figure 5. Counter-rotating spinner pair. A) Distance between counter-rotating spinners as a function of Re. B) Translational velocity of the center of mass of the counter-rotating pair along the direction orthogonal to the vector joining both centers at Re = 0.18 (black lines), 0.46 (red lines), and 0.84 (blue lines).

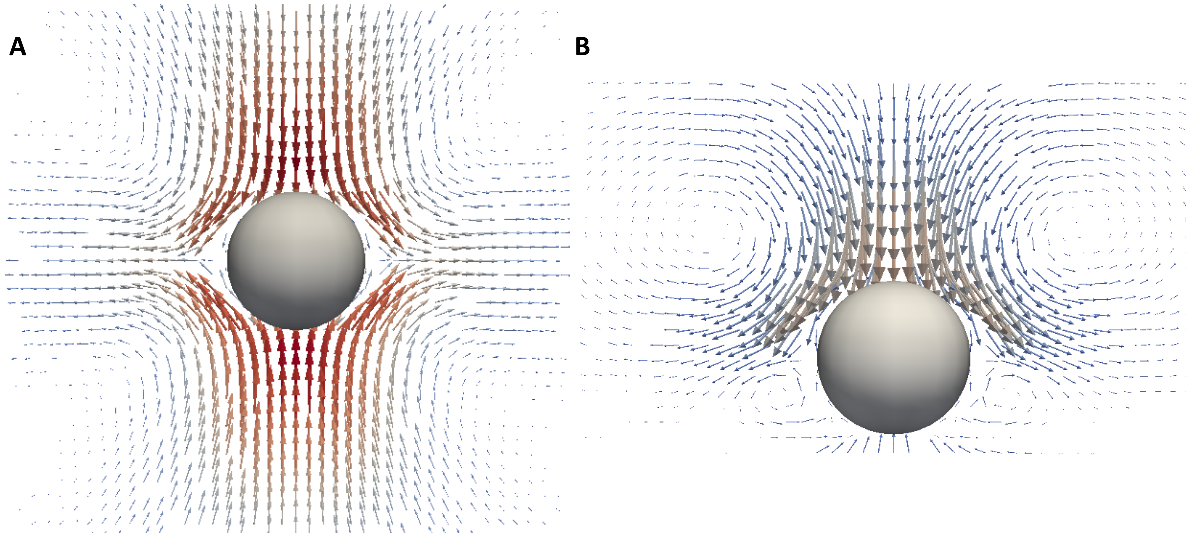

Supplementary Figure 6. Secondary flow. Secondary flow in the xz plane generated by a spinner rotating about the z-axis in bulk (A) and close to a wall (B)

.

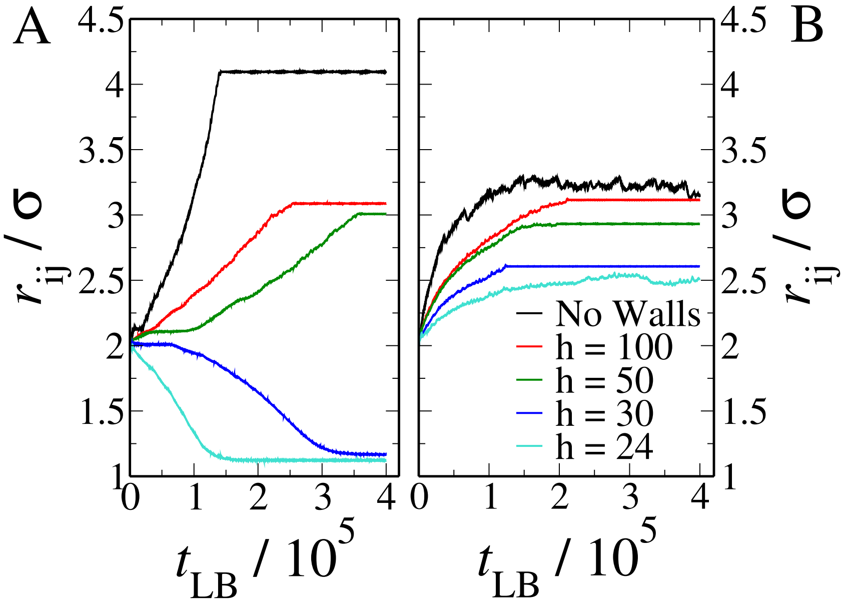

Supplementary Figure 7. Wall effects. A) Time evolution of the distance between counter-rotating spinners in absence of walls (black line) and confined in channels with different heights: h=100 (red line), h=50 (green line), h=30 (blue line), and h=24 (turquoise). B) Time evolution of the distance between co-rotating spinners in absence of walls (black line) and confined in channels with different heights: h=100 (red line), h=50 (green line), h=30 (blue line), and h=24 (turquoise).

.

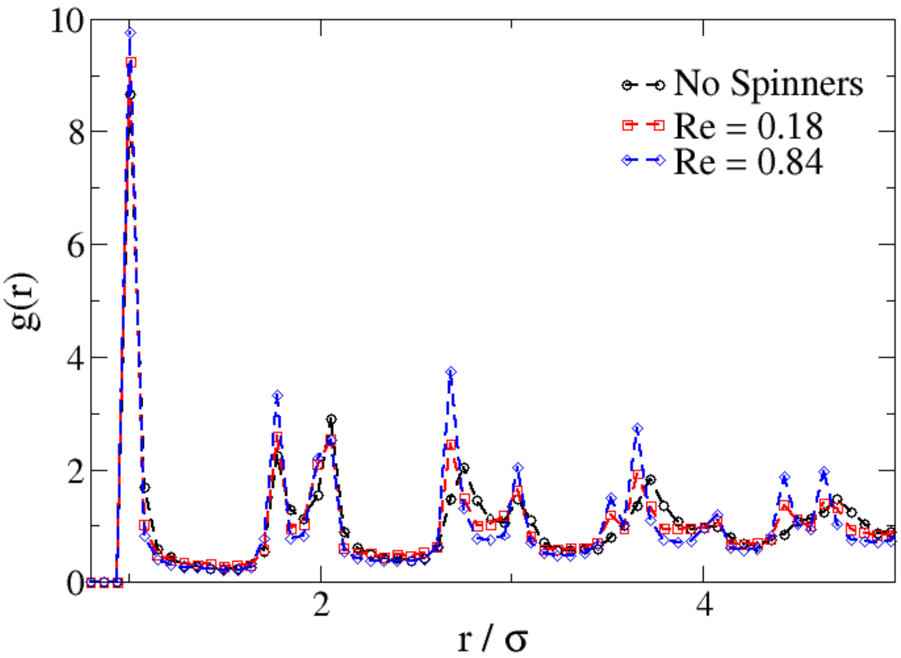

Supplementary Figure 8. Radial distribution function. Two-dimensional radial distribution function for the hard-sphere monolayer in the absence of spinners (black circles) and in the presence of a spinner rotating at $Re = 0.46$ (red squares) and $Re = 0.84$ (blue diamonds).

.

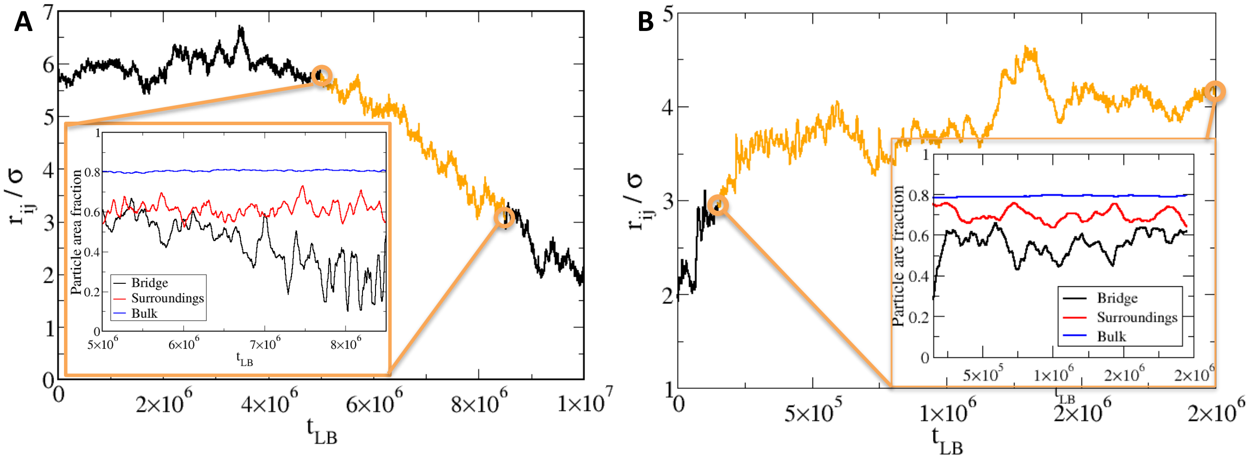

Supplementary Figure 9. Evolution of the distance between spinners embedded in a passive particle monolayer. A) Time evolution of the distance between two co-rotating spinners embedded in a passive monolayer of $\phi = 0.8$ using numerical simulations at $Re = 0.84$. Inset: Time evolution of the particle area fraction of the bridge (black line), the surroundings (red line), and the bulk (blue line). B) Time evolution of the distance between two counter-rotating spinners embedded in a passive monolayer of $\phi = 0.8$ using numerical simulations at $Re = 0.84$. Inset: Time evolution of the particle area fraction of the bridge (black line), the surroundings (red line), and the bulk (blue line).

.

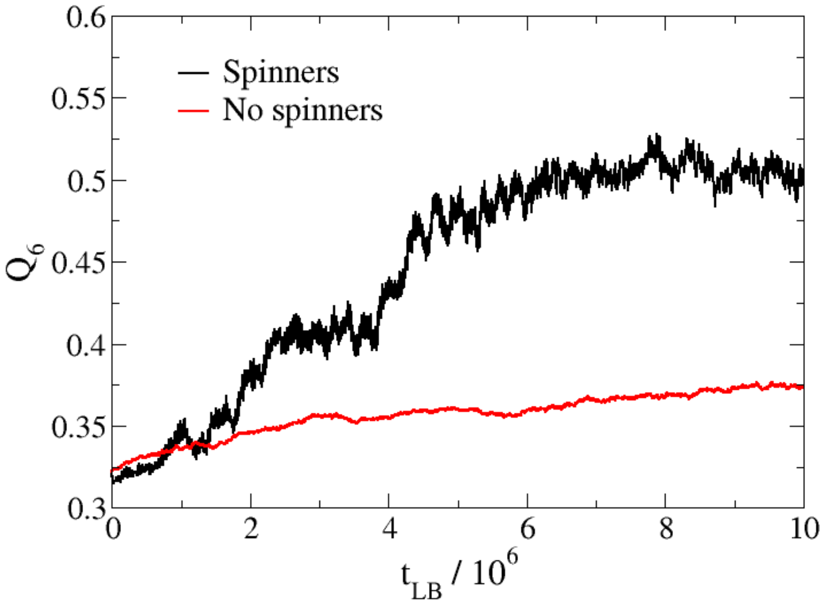

Supplementary Figure 10. Bond orientational order parameter. Average order, quantified by Q_6, of two passive monolayers at $\phi = 0.8$: i) In absence of spinners (red line) and ii) in the presence of two spinners (black line) at $Re = 0.84$.

.

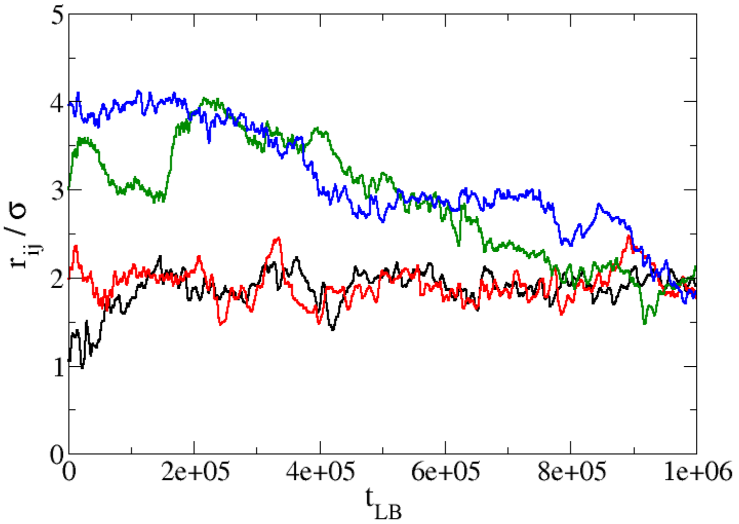

Supplementary Figure 11. Evolution of the distance between spinners embedded in a hexagonal lattice of passive particles. Time evolution of the distance between two co-rotating spinners at $Re = 0.84$ using as initial configuration a perfect hexagonal lattice at $\phi = 0.8$.

.

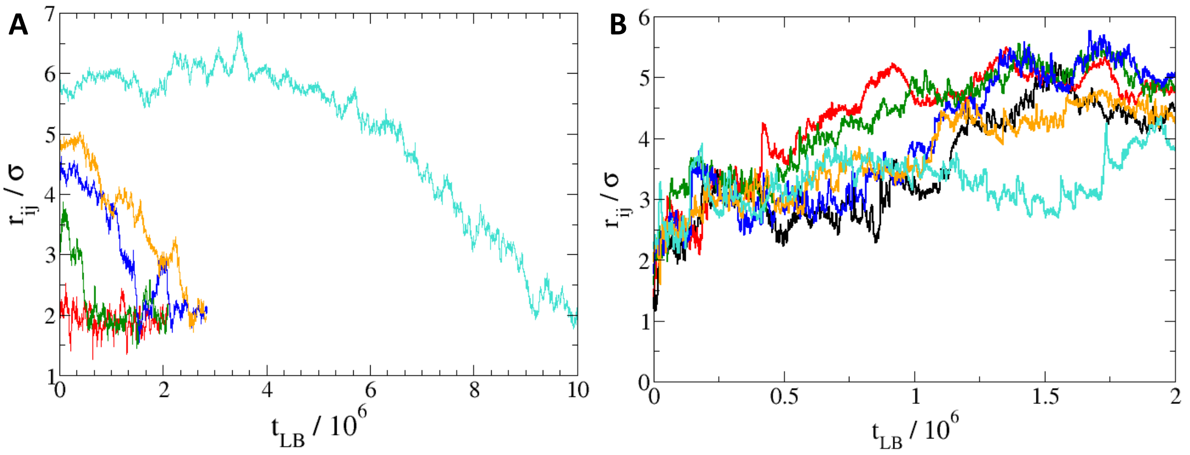

Supplementary Figure 12. **Evolution of the distance between spinners embedded in a passive particle monolayer**. Simulated time evolution of the distance between two co-rotating (A) and two counter-rotating (B) spinners at $Re = 0.84$ for spinners initially positioned at different distances. The monolayer area fraction is of 0.8.

.

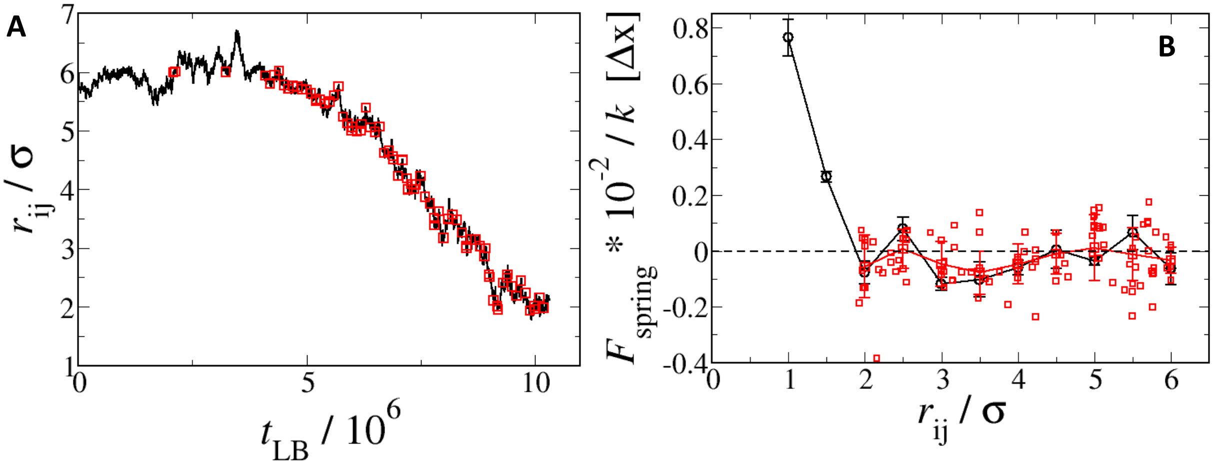

Supplementary Figure 13. Spinner-Spinner interaction within passive monolayers of $\phi_A = 0.8$. A) Trajectory of attraction between to co-rotating spinners embedded in a passive monolayer of $\phi_A = 0.8$. The red squares correspond to the selected configurations to compute the force by using harmonic springs. B) Spring forces as a function of the separation distance between co-rotating spinners. The black circles represents the average force over five independent initial configurations, while the small red squares correspond to the spring forces measured for the selected configurations taken from the trajectory of Supplementary Figure 13. The big red squares represent the average over the selected configurations as a function of the distance.

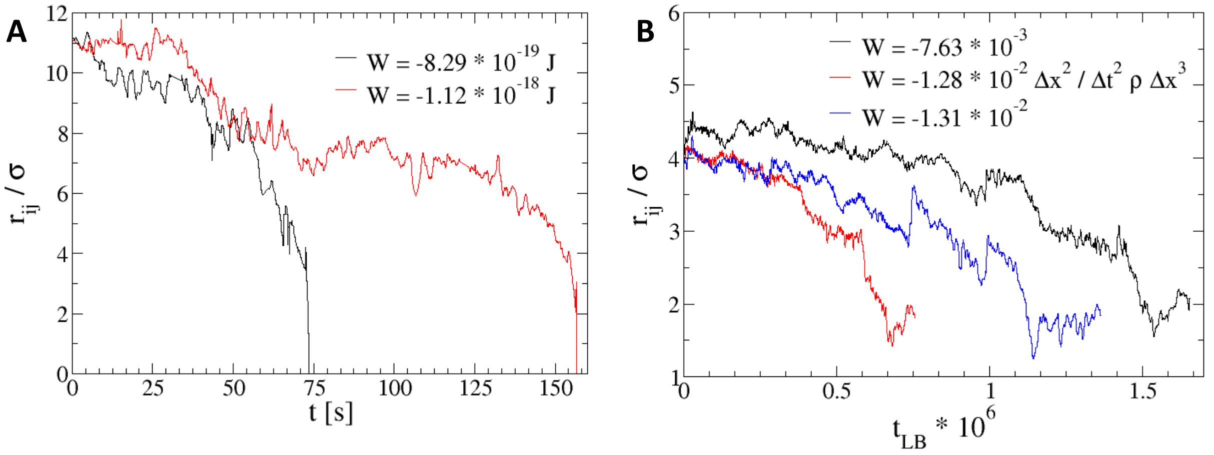

Supplementary Figure 14. Work done by the spinners on the fluid along attractive trajectories. A) Experimental and B) Simulation trajectories of the time evolution of the distance between co-rotating spinners in passive monolayers of about ϕ_A =0.7 and $\phi_A = 0.8$, respectively.

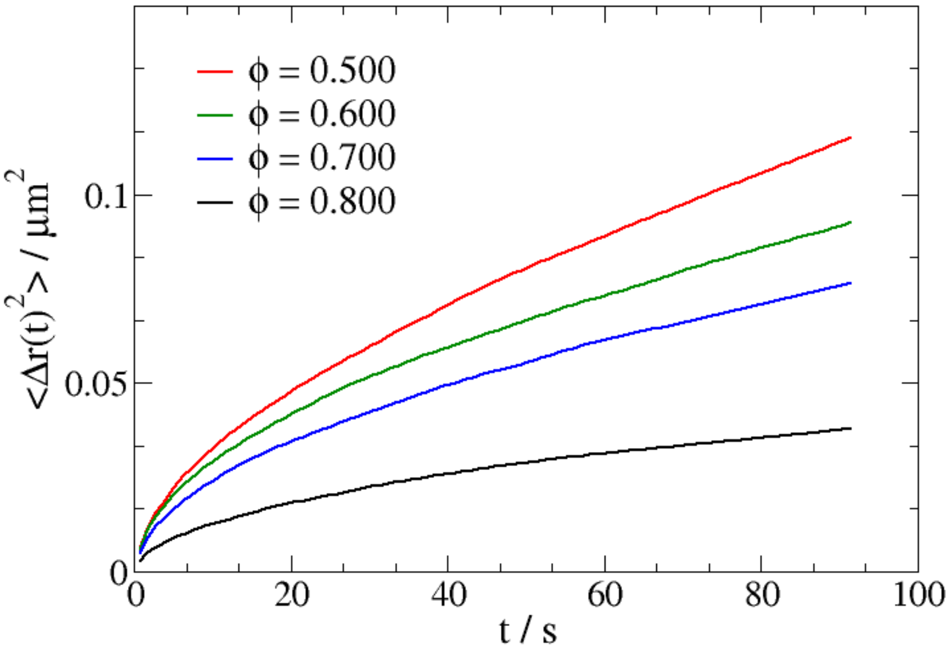

Supplementary Figure 15. Mean square displacements. Time-dependent mean square displacement, $\langle \Delta r^2(t) \rangle$, of pseudo hard-sphere monolayers at area fractions of $\phi = 0.8$ (black), $\phi = 0.7$ (blue), $\phi = 0.6$ (green) and $\phi = 0.5$ (red). To transform from Lattice-Boltzmann units to real units we have calculated that $\Delta x = 0.42\mu$m and $\Delta t = 7.14310^{-3}$ s.

.

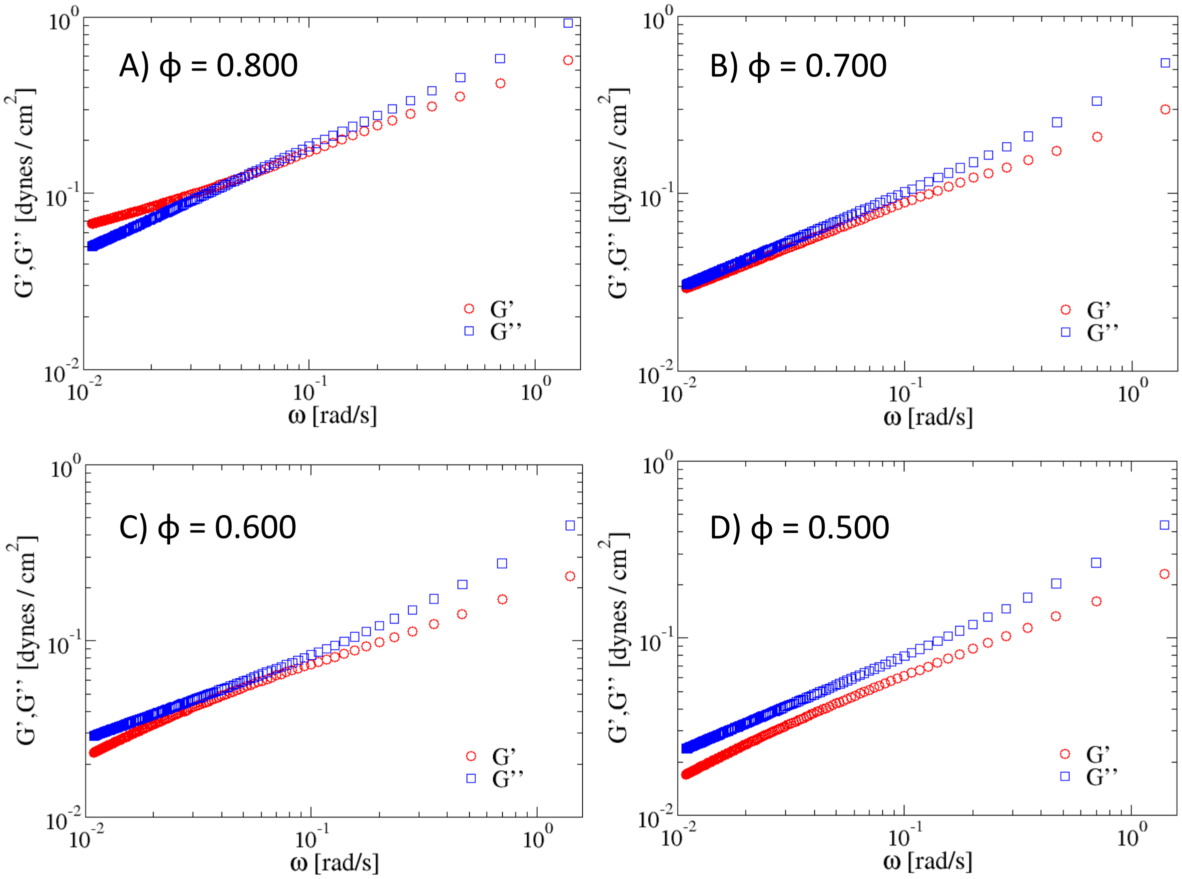

Supplementary Figure 16. Frequency-dependent viscoelastic modulus. Frequency-dependent storage modulus (red circles), $G'(\omega)$, and loss modulus (blue squares), $G''(\omega)$, for passive monolayers at area fractions of $\phi = 0.8$ (A), $\phi = 0.7$ (B), $\phi = 0.6$ (C) and $\phi = 0.5$ (D).

.

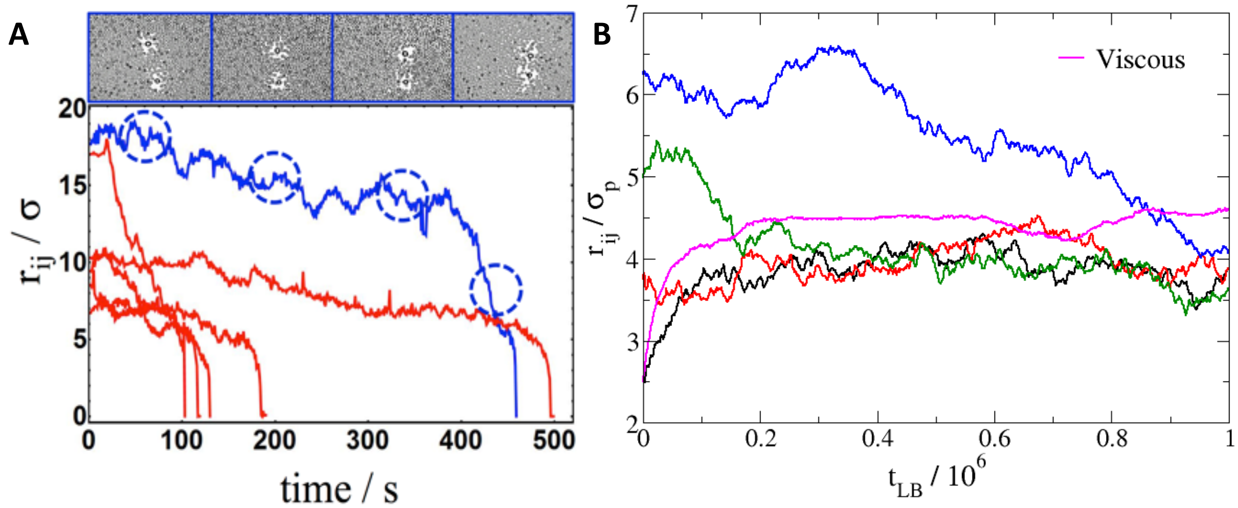

Supplementary Figure 17. Effect of the relative particle size on the spinner-spinner interaction. A) Experimental trajectories of the distance between spinners of the same size as the passive particles red lines), $\sigma_a = \sigma_p = 5$ μm, and for two spinners larger than the passive particles (blue line), $\sigma_a = 9$ μm and $\sigma_p = 5$ μm. B) Simulation trajectories of the distance between spinners of diameter $\sigma_a = 1.5$ σ_p at different initial distances. The magenta line represents the repulsion trajectory of two spinners in the absence of passive particles. For distances larger than the “equilibrium distance” there is a net attraction (green and blue curves).

.

II. SUPPLEMENTARY DISCUSSION

A. Fluid Field Generated by a Rotating Spherical Particle

A spinner suspended on a viscous fluid and rotating around the z-axis at a frequency ω generates a rotating fluid flow, which velocity field is given by Eq. 2 at $Re = 0$. In spherical polar coordinates (r, θ, ϕ), the azimuthal fluid velocity decays as $\frac{1}{r^2}$, as shown in Supplementary Figure 2A. This fluid velocity contribution is the only contribution at $Re = 0$ and is independent of the Re, as also shown in Fig. 2A. However, at small but finite Re an additional polar contribution to the fluid velocity appears originated by inertial effects, so-called secondary flow, and which therefore depends on the Re, as demonstrated in Supplementary Figure 2B. This described scenario corresponds to spinners suspended on a fluid and far from a wall. The presence of a wall modifies such scenario as the fluid velocity, azimuthal and polar, decays faster that in the absence of walls, as $\frac{1}{r^3}$, as shown in Supplementary Figure 2A and B. This type of confinement effect has been already described [1].

The presence of a wall breaks the symmetry of the secondary flows generated by the spinner rotation and also reduces the magnitude of the secondary flow, which translates into a depletion of the inertial effects for spinners close to a wall as discussed in the main text. In Supplementary Figure 4 the profiles of the azimuthal and polar fluid velocities are presented as a function of the channel height for a spinner rotating at Re=0.6. The azimuthal fluid velocity component is barely affected by the channel size. On the contrary, the strength of the secondary flow is significantly reduced by narrowing the channel, which results in a shorter repulsion distance for smaller channels, as shown in Supplementary Figure 7.

In these simulations the simulation box was discretized in three dimensional grids with resolution $N_x \times N_y \times N_z = 220 \times 220 \times 200$ and periodic boundary conditions for the simulations in the absence of walls or $N_x \times N_y \times N_z = 220 \times 220 \times 30$ bounded in the z direction by no-slip walls and periodic boundary conditions in the x and y directions for the simulations in the presence of a channel. The rest of simulation details were the same as the ones described in the main text.

B. Counter-Rotating Spinners in a Viscous Fluid

When suspended on a viscous fluid, counter-rotating spinners separated by 2σ or less show an effective attraction, thereby forming a doublet that moves along the direction orthogonal to the vector joining both centers [2]. The equilibrium distance between both spinners is independent on Re, as shown in Supplementary Figure 5A; however, the strength of the interaction, given by the slope of the attraction trajectory, and the translational velocity of the doublet do, Supplementary Figure 5B. This points out that the interaction between counter-rotating spinners suspended in a viscous fluid is of an inertial nature.

C. Secondary Flow Generated by the Spinner Rotation

At small but finite Re an additional polar contribution to the fluid velocity appears originated by inertial effects, the profile of the secondary flow in the presence and absence of a wall are represented in Supplementary Figure 6.

D. Channel Effect

Decreasing the height of the channel leads to a reduction of the hydrodynamic repulsion between co-rotating spinners due to the reduction of the lift forces exerted on the spinners. As discussed above, the presence of a channel weakens the strength of the secondary flow, such that it is reduced by shortening the channel height. In the case of counter-rotating spinners, this depletion of the lift forces leads to the switching of the interaction from repulsive to attractive for channel heights smaller than h=50, as shown in Supplementary Figure 7.

E. Radial Distribution Functions

The two-dimensional radial distribution function (RDF) of the monolayer, represented in Supplementary Figure 8, shows that the spinner produces a compression of the passive monolayer, as well as an increase in the order of thereof, which is also shown by the bond-orientational order parameter, plotted in Supplementary Figure 10. The fluid flows generated

by the spinners produce a depletion region around them, compressing the passive monolayer, which in turn contributes to its arrangement.

F. Time Evolution of the Particle Area Fraction from the Different Regions of the System

The compression and shear stresses produced by co-rotating spinners in the bridge, through the corona, result in a density reduction within this region as it is constantly yielding due to shear stresses induced by the spinners. Thus, the higher mobility of passive particles initially located in this region allows them to migrate to less stressed regions. On the contrary, for counter-rotating spinners, the density of passive particles in both the bridge and surroundings is similar, as presented in Figs. 9A and B. Figs. 9A and B are the counterparts of Fig. 5C and D in the main text, in which the position of the spinners is let free to evolve.

G. Annealing of the Passive Monolayer

To probe the annealing of the passive monolayer produced by the spinners we quantify the degree of order calculating for each passive particle the 2D local bond-orientational order parameter Q_6. Bond-orientational parameters were introduced to measure the local structure around a particle [3, 4]. Bonds are defined as the vector joining a pair of neighboring particles, $\mathbf{r}_{ij}$, where we define neighboring particles as those which are at a shorter distance than the first minimum of the RDF. Once we have identified the neighbors of each particle, we define a $(2l+1)$ dimensional complex vector for each particle with the components:

$$q_{lm}(i) = \frac{1}{N_b(i)} \sum_{j=1}^{N_b(i)} Y_{lm}(\mathbf{r}_{ij}) \tag{1}$$

where $N_b(i)$ corresponds to the number of neighboring particles of particle i, $Y_{lm}(\mathbf{r}_{ij})$ are spherical harmonics evaluated for the direction of the bond, determined by the azimuthal angle ϕ_{ij}. To make the order parameters invariant with respect to rotations of the reference frame, the second-order invariants are defined as

$$q_l(i) = \left(\frac{4\pi}{2l+1} \sum_{m=-l}^{l} |q_{lm}(i)|^2 \right)^{1/2} \tag{2}$$

The global order parameter is obtained by averaging over all bonds in the system:

$$Q_l = \left\langle \frac{1}{N_b} \sum_{j=1}^{N_b} e^{li\phi_{ij}} \right\rangle \tag{3}$$

The bond-orientational order parameter Q_6, averaged over all bonds, is used to monitor the global structural changes of the system, as shown in Supplementary Figure 10. In a fully disordered state, i.e. fluid, we will observe vanishing values of the bond order parameters. By contrast, the more ordered the system, the higher the value of the global bond order parameter. Therefore, the presence of the spinners promote the ordering of the passive monolayer.

H. Initial Configuration: Perfect HCP

To discard the migration and coalescence of defects as the diving force for the co-rotating spinners attraction exhibited in dense passive monolayers we performed simulations in which the initial configuration of the monolayer was a perfect hexagonal close packing lattice (hcp). We monitored the time evolution of the distance between two co-rotating spinners in a perfect hcp lattice, and observed that they come close together, even in the absence of defects, as a result of the effective attraction induced by the passive monolayer, as shown in Supplementary Figure 11.

I. Co- and counter-rotating spinners in passive monolayers

The effective interaction between two spinners embedded in a dense passive monolayer is of stochastic nature; it depends on the instantaneous configuration of the passive monolayer. The timescale of rearrangement of the monolayer in the vicinity of the active beads is in the same order as the characteristic timescale of the motion of the active beads themselves because they are intimately coupled. In fact, the dynamic trajectories of the distance between the spinners initially positioned at different distances show an almost linear regime of attraction under for some times, as well as lag times, as it can be seen in Fig. 12. This means that during the trajectory the spinners approach to each other at a constant speed on average. Assuming a Stokes' scenario ($F = 6\pi\mu\sigma/2U$) for the translation of the spinners through the monolayer along their attraction trajectory, the strength of this interaction

would be a constant and independent of the distance between them.

J. Spinner-Spinner interaction in passive monolayers

We evaluate the interaction potential between a co- and counter-rotating spinner pair embedded in a passive monolayers of $\phi_A = 0.8$ by restraining the distance between them by means of harmonic potentials, $F = k(r - r_0)$, at different separation distances, r_0. From an disordered monolayer of $\phi_A = 0.8$ we randomly select a pair of particles separated by a distances r_0 and labeled them as spinners. We chose five independent spinner pairs at each separation distance r_0 in the range of 1σ to 6σ at intervals of 0.5σ. We allow the system to relax and then we sample over 8000 thousand configurations the spring force as a function of the distance displacements. Then, by performing the Riemann integral of that curve we compute the potential of mean force (PMF), W, at each separation distance r_0. The mean spinner-spinner interaction potential is then obtained by averaging over the five independent configurations at each separation distance, as shown in Fig. 6. Therefore, if the PMF is greater than 0, the interaction due to the media between the spinners is repulsive, while if it is negative the spinner-spinner interaction is attractive.

As discussed in the main text, the force exerted on the spinners by the media or spinner-spinner interaction potential strongly depends on the instantaneous configuration of the monolayer. This is intimately related with the fact that the spinners need to load the bridge before it yields, which results in the approaching of co-rotating spinners. The amount of stress needed to load the bridge above that threshold obviously depends on the bridge/monolayer configuration. To proof this we compute the spring forces as a function of the separation distance between co-rotating spinners for 100 configurations taken from the simulation trajectory of co-rotating spinners initially separated by a distance of 6σ presented in Fig. 4, as shown in Supplementary Figure 13A. One can clearly see that the forces acting on the spinners, and thus, the force exerted by the spring on the spinners, strongly depends on the configuration of the monolayer. Interestingly, by averaging the spring forces as a function of the separation distance between the spinners (red line in Supplementary Figure 13B), we observe that the forces acting on the spinners follow a similar trend than the ones computed by averaging over 5 independent initial configurations of the monolayer (black line in Supplementary Figure 13B), as shown in Supplementary Figure 13B. This means

that although we can not predict the exact interaction between two spinners separated by a certain distance because the exact strength of the interaction depends on the instantaneous configuration of the system, and not on the separation distance, we can confidently say that the interaction between two co-rotating spinners is attractive.

As mentioned before, the emergent spinner-spinner interaction in the presence of a passive matrix is of a stochastic nature. To show this more clearly, we have computed the work done by co-rotating spinners on the fluid, with which they are in contact, along some experimental and simulation trajectories that exhibit spinner-spinner attraction, such as the ones shown in Supplementary Figure 14. Note that the spinners are typically only in contact with the fluid and they transmit all the stresses through it. From those trajectories we computed the approaching velocity of the spinners (U) along their "reaction coordinate", which is the line connecting them. Assuming a Stoke's scenario, the work can be calculated by multiplying the force exerted by spinner translation on the surrounding fluid ($F = \gamma U$). Then, from the force vs distance curve we computed the work done by the spinners on the fluid, or vice versa, the work done by the fluid on the spinners. This only depends on the sign of the forces. As can be seen in Supplementary Figure 14, different trajectories of co-rotating spinners initially positioned at similar distances show different amount of dissipation (work done by the spinners on the fluid). This proves the stochastic nature of this interaction and reflects its dependence on the monolayer configuration. In fact, if an emergent interaction potential between the spinners embedded in passive monolayers did in fact exist, then this interaction would be deterministic and the work done by the spinners on the surrounding fluid would be the same for all the trajectories starting at point A and ending up at point B. The non-equilibrium nature of this system makes that the spinner-spinner interaction within passive monolayers an activated process and thus, stochastic. As already explained, the spinners need to build up stresses on the bridge before it yields, which obviously depends on the instantaneous configuration of the bridge/monolayer.

K. Mechanical properties of the passive monolayer

To further investigate the origin of the force reversal between spinners in the presence of the passive matrix, we study the mechanical properties of the system by using our simulation model and experiment. We measure the time evolution of the mean square displacement,

MSD, of the passive particles in monolayers at different area packing fractions. The MSD contains information about the mechanical properties of the material; in pure viscous materials the MSD varies linearly with time, where the slope of the curve is determined by the diffusion coefficient, D. However, in solid-like materials the MSD reaches a characteristic plateau, which is related to the elastic energy of the system [5–7]. We observe that for high packing fractions the MSD deviates from the linear behavior (see Supplementary Figure 15), which indicate the solid-like character of the hard-sphere monolayer at packing fractions above 0.6 [8]. By contrast, in the absence of passive particles, the system behaves as a viscous fluid.

From the time dependent mean square displacement, $\langle \Delta r^2(t) \rangle$, we obtain $G^*(\omega)$ from a frequency dependent form of the Stokes-Einstein equation. Although this relationship breaks for glasses in which hopping occurs [9], we have checked that this type of events does not take place during the simulated trajectories from which we computed the MSD. Hence, we can assume that each passive particle of the monolayer feels an isotropic, incompressible continuum, and the viscoelastic spectrum of the medium, and compute the MSD of the probe particle as the average displacement of all the particles in the system. The complex shear modulus is then computed using an algebraic form of the generalized Stokes-Einstein equation [10–12],

$$G^*(\omega) = \frac{k_B T}{\pi a \left\langle \Delta r^2(1/\omega) \right\rangle \Gamma \left[1 + \left(\partial ln \left\langle \Delta r^2(1/\omega) \right\rangle / \partial(1/\omega)\right)\right] \left[1 + \left(\partial^2 ln \left\langle \Delta r^2(1/\omega) \right\rangle / \partial(1/\omega)^2\right)\right]} \tag{4}$$

where a is the particle radii, Γ is the gamma function and ω is the frequency. First, the MSD data is fitted to a second-order polynomial function from which the first and second time derivatives of the MSD are computed, and then $G^*(\omega)$ is computed following Eq. 4. The storage and loss moduli are obtained by fitting $G^*(\omega)$ to a power-law and solving the following equations, respectively:

$$G'(\omega) = G^*(\omega) \left(\frac{1}{[1 + \beta(\omega)]} \right) cos \left[\frac{\pi}{2} \alpha(\omega) - \beta(\omega)\alpha(\omega) \left(\frac{\pi}{2} - 1 \right) \right] \tag{5}$$

$$G''(\omega) = G^*(\omega) \left(\frac{1}{[1 + \beta(\omega)]} \right) sin \left[\frac{\pi}{2} \alpha(\omega) - \beta(\omega)[1 - \alpha(\omega)] \left(\frac{\pi}{2} - 1 \right) \right] \tag{6}$$

where $\alpha = (\partial ln \left\langle \Delta r^2(1/\omega) \right\rangle / \partial \omega)$ and $\beta = (\partial^2 ln \left\langle \Delta r^2(1/\omega) \right\rangle / \partial \omega^2)$.

The computed storage and lost moduli for different packing fractions are presented in Supplementary Figure 16. These results show that below packing fractions of about 0.7 the elastic response of the system drops below the viscous response and hence, the system behaves as a viscous material. Whereas for packing fractions above 0.7 the system behaves as a solid-like material.

L. Effect of the size ration between active and passive particles

To test the effect of the size ratio between active and passive particles, we perform experiments using bigger spinners ($\sigma_a = 9$ μm against the $\sigma_p = 5$ μm of the passive particles). We do not observe differences in the range or strength of the interaction between spinners as a function of the size ratio between the active and passive particles, as shown in Supplementary Figure 17A. In addition, we also perform simulations in which the spinners are 1.5 larger than the passive particles. In agreement with the experimental observations, we do not observe significant differences in the interaction object of this study, as shown in Supplementary Figure 17B.

III. SUPPLEMENTARY METHODS

A. Sample Preparation

Ferromagnetic particles, approximately 5μm in diameter provided by Spherotech and henceforth referred to as spinners, were diluted 2000$\times$ in a surfactant solution. 10μL of this solution was mixed with 40μL of the surfactant solution. The solution was vortexted for several minutes and then 10μL of this solution was inserted into a channel. The channels were created using glass slides and strips of 3M double sided tape. Three pieces of tape were placed on top of each other and then small strips were cut and then placed on the slide to make channels approximately 22$\times$3mm rectangular channels. Once the solution was inserted the channel was sealed with epoxy to prevent evaporation and evaporative induced flows. The slide was placed atop a neodymium magnet to magnetize the spinners and then moved to the sample holder. After several minutes, to allow for sedimentation, the magnetic field was actuated for approximately five minutes at a frequency of 5 Hz.

To investigate the interaction between spinners in the presence of a passive medium we doped a monolayer of passive particles with ferromagnetic particles. Ferromagnetic particles were again diluted 2000$\times$ in a surfactant solution. 10μL of this solution was mixed with 40μL of polystyrene particles, approximately 5μm in diameter provided by Phosphorex. This solution was vortexted for several minutes, and then 10μL was inserted into the channel. The slide was placed atop a neodymium magnet to magnetize the spinners and then moved to the sample holder. After several minutes, to allow for sedimentation, the packing fraction was determined to be approximately 0.7, and the magnetic field was actuated for approximately five minutes at a frequency of 5 Hz.

B. Experimental Apparatus

To drive the spinners we developed the experimental apparatus seen in Supplementary Figure 1. The apparatus consists of two pairs of coils attached to a rectangular wooden box. The wooden box was mounted on a modified compound binocular light microscope (condenser lens, iris diaphragm, stage, condenser focus all removed) provided by OMAX. Another OMAX microscope was modified so that it would act as a 3D mobile stage. The box was then mounted so that the coils were centered on the sample holder. Two sinusoidal signals, phase shifted by 90 degrees, were generated by an external program, Audacity, and passed through each pair of coils (one signal per pair of coils). A 300W amplifier (150W/channel) ran 2.5 Amps through more than 100 turns of wire (in each coil) to obtain a field strength of approximately 5mT.

IV. SUPPLEMENTARY REFERENCES

---

[1] Lubensky DK, Goldstein RE. Hydrodynamics of monolayer domains at the air–water interface. Physics of Fluids. 1996;8(4):843.

[2] Leoni M, Liverpool TB. Dynamics and interactions of active rotors. Europhysics Letters. 2011 Jan;92(6):64004–p1–64004–p6.

[3] Wang Y, Teitel S, Dellago C. Melting of icosahedral gold nanoclusters from molecular dynamics simulations. Journal of Chemical Physics. 2005 Jun;122(2):214722–214722.

[4] Steinhardt PJ, Nelson DR, Ronchetti M. Bond-orientational order in liquids and glasses. Physical Review B (Condensed Matter). 1983 Jul;28(2):784–805.

[5] Dasgupta B, Tee SY, Crocker J, Frisken B, Weitz D. Microrheology of polyethylene oxide using diffusing wave spectroscopy and single scattering. Physical Review E. 2002 May;65(5).

[6] Mason T, Weitz D. Optical measurements of frequency-dependent linear viscoelastic moduli of complex fluids. Physical Review Letters. 1995 Feb;74(7):1250–1253.

[7] Squires TM, Mason TG. Fluid Mechanics of Microrheology. Annual Review of Fluid Mechanics. 2010 Jan;42(1):413–438.

[8] Mason T, Weitz D. Linear viscoelasticity of colloidal hard sphere suspensions near the glass transition. Physical Review Letters. 1995 Oct;75(14):2770–2773.

[9] Charbonneau P, Jin Y, Parisi G, Zamponi F. Hopping and the Stokes-Einstein relation breakdown in simple glass formers. Proceedings of the National Academy of Sciences. 2014 Oct;111(42):15025–15030.

[10] Crocker JC, Valentine MT, Weeks ER, Gisler T, Kaplan PD, Yodh AG, et al. Two-point microrheology of inhomogeneous soft materials. Phys Rev Lett. 2000 Jul;85(4):888–891.

[11] Dasgupta BR, Weitz DA. Microrheology of cross-linked polyacrylamide networks. Physical review E, Statistical, nonlinear, and soft matter physics. 2005 Feb;71(2):021504.

[12] Mason TG. Estimating the viscoelastic moduli of complex fluids using the generalized Stokes–Einstein equation. Rheologica acta. 2000;39(4):371–378.
